# Serum Brain-Derived Neurotrophic Factor: An Emerging Marker for Diabetic Retinopathy

**DOI:** 10.7759/cureus.74153

**Published:** 2024-11-21

**Authors:** Vivek Ram K. U., Sheelu S Siddiqi, Tanusree Debbarman, Amit Mukherjee, Naheed Akhtar, Abdul Waris, Dimple Thacker, Shaista

**Affiliations:** 1 Institute of Ophthalmology, Jawaharlal Nehru Medical College and Hospital, Aligarh Muslim University (AMU), Aligarh, IND; 2 Rajiv Gandhi Centre for Diabetes and Endocrinology, Jawaharlal Nehru Medical College and Hospital, Aligarh Muslim University (AMU), Aligarh, IND

**Keywords:** brain-derived neurotrophic factor (bdnf), diabetes type 2, diabetic retinopathy, hyperglycemia, neurotrophin

## Abstract

Background: Diabetes mellitus (DM) is a global disease that is strongly associated with both microvascular and macrovascular complications. A significant proportion of individuals with diabetes develop diabetic retinopathy (DR), a microvascular complication that can lead to blindness, particularly in working-age adults. Diabetes adversely affects the entire neurosensory retina, with accelerated neuronal apoptosis and activation or altered metabolism of neuroretinal supporting cells. These findings suggest that DR could be a sensory neuropathy that affects the retinal parenchyma, similar to peripheral diabetic neuropathy. Neurotrophins have been implicated in the progression of DR. This study was done to determine the association of serum brain-derived neurotrophic factor (BDNF) levels with DR in type 2 diabetic patients in a tertiary care hospital in North India.

Methods: A hospital-based case-control study was done. The case group included diabetic patients with retinopathy (n=44) and the control group included diabetic patients without retinopathy (n=44). Serum BDNF levels were estimated by enzyme-linked immunosorbent assay (ELISA). Statistical analysis was carried out using GraphPad Prism software (Dotmatics, Boston, USA).

Results: The mean serum BDNF in the control and case groups was 2753 ± 465 pg/ml and 1598 ± 483 pg/ml, respectively. The difference between the two groups was statistically significant as per the Mann-Whitney U test (U=64, p<0.0001). Logistic regression analysis showed that BDNF is a good predictor for DR after multivariate regression.

Conclusions: Patients with DR were found to have lower serum BDNF levels compared to those without retinopathy. BDNF may serve as an early predictor for DR. Due to its role in neuronal health and metabolism, increasing BDNF levels could offer a therapeutic approach to managing diabetes-related complications. However, further research is needed to confirm the effectiveness of BDNF in slowing or preventing the progression of DR.

## Introduction

Diabetes mellitus (DM) is a global disease. The tenth edition of Diabetes Atlas by the International Diabetes Federation (IDF) released in 2021 estimated that there are 537 million persons with diabetes worldwide. According to IDF data for 2021, there are nearly 74 million cases of adult diabetes in India with a prevalence of 8.3% [1]. Diabetes has many complications - retinopathy being one among them. Diabetic retinopathy (DR) is a progressive condition that impacts the blood vessels in the retina. It is a widespread complication of diabetes globally, affecting approximately 35% of individuals with the disease. The most severe consequence of DR is blindness [2].

Brain-derived neurotrophic factor (BDNF) in DM

BDNF is a protective factor that has a role in neuronal growth, survival, and synaptic adaptability and regulates the synthesis and uptake of neurotransmitters. Reduced levels of BDNF in type 2 diabetes are caused by a combination of chronic hyperglycemia, which leads to oxidative stress and advanced glycation end products (AGEs) formation, insulin resistance that disrupts BDNF signaling, and inflammation from cytokines like TNF-α and IL-6, which inhibits BDNF expression and neurogenesis [3-5]. Epigenetic changes, such as DNA methylation triggered by metabolic disturbances, further reduce BDNF production [6]. Additionally, stress and depression, common in diabetes, lower BDNF through cortisol, while sedentary lifestyles and poor diets exacerbate the decline [7,8]. Addressing these factors may help restore BDNF levels and improve diabetes outcomes.

BDNF and DR

The reduced expression of BDNF in the retina may contribute to retinal ganglion cell apoptosis, leading to the progression of DR. Research is ongoing to explore whether BDNF supplementation could have therapeutic effects in preventing or slowing down the progression of this condition [3]. Our research aimed to find the correlation between serum BDNF and DR. There are hardly any studies done on this subject in this part of the world. Also, existing literature doesn’t give a clear picture of BDNF and its role in DR. Hence, we took up this study to enhance our understanding of this molecule and to look for the possibility of using it as a diagnostic marker in DR.

## Materials and methods

A hospital-based case-control study was conducted in a tertiary care center in North India. The study was started after obtaining clearance from the Institutional Ethics Committee (Ref No: IECJNMC/880) and was done strictly in accordance with the Declaration of Helsinki. The study duration was from October 2022 to August 2024.

Study subjects

A total of 88 subjects were enrolled in the study after obtaining informed written consent. All subjects recruited for this study had type 2 DM of which 44 had retinopathy (n=44) and 44 were without retinopathy (n=44). Type 2 DM subjects were diagnosed according to the current updated criteria of the American Diabetes Association (ADA). Individuals who were willing to participate and adhere to the study protocol were only included. Patients with type 1 DM, cardiovascular diseases, diabetic nephropathy, or patients who had received prior anti-vascular endothelial growth factor (VEGF) injections/laser photocoagulations were excluded from the study. A detailed proforma of the patient was filled out, wherein all relevant socio-demographic and personal details of the patient were recorded.

Ophthalmological examination

A dilated fundus examination was performed using an indirect ophthalmoscope (Keeler, UK) and a 20D lens (Volk Optical, USA) to diagnose DR. Fundus photographs of both eyes were captured with Visucam 500 (Carl Zeiss Meditec, Jena, Germany) and used to grade DR based on the early treatment diabetic retinopathy study (ETDRS) grading criteria.

Blood sample collection

A 5 ml blood sample was collected from each participant using a gel clot-activated vial. Following centrifugation at 1500 rpm for five minutes, 3 ml of serum was transferred into an Eppendorf tube for immediate biochemical analysis, while the remaining 2 ml was set aside for BDNF measurement and stored at -20°C in a Vestfrost freezer (Denmark) until further analysis.

Biochemical investigations

Fasting blood glucose, postprandial blood glucose, lipid profile, and renal function were evaluated using a Beckman Coulter AU480 fully automated analyzer (Beckman Coulter Inc., California, USA) with commercially available kits from Beckman Coulter. Glycated hemoglobin (HbA1c) levels were determined through the high-performance liquid chromatography (HPLC) method with the Premier Hb9210 analyzer (Trinity Biotech, Bray, Ireland).

Serum BDNF estimation

Serum BDNF was estimated using GENLISA ELISA kits (Ref: KBH1302) by Krishgen Biosystems (India). The standard calibration range of the kit was 31.25-2000 pg/ml.

Statistical analysis

Data was analyzed using the latest GraphPad Prism software version 10 (Dotmatics, Boston, USA) and expressed as mean ± SD or 95% confidence interval. Proper statistical analysis was performed after performing the normality test. A value of p<0.05 was considered a statistically significant difference.

## Results

The comparison of demographic, physical, and biochemical characteristics between the two study groups is shown in Table 1. There was no statistically significant difference in age, blood pressure, serum hemoglobin A1c (HbA1c), and fasting blood sugar (FBS). However, a statistically significant difference was seen in sex ratio, duration of diabetes, post-prandial blood sugar, blood urea nitrogen (BUN), serum creatinine, and serum BDNF.

**Table 1:** Demographic, physical, and biochemical characteristics of 88 patients included in the study. *Independent T test; **Fisher's exact test; ***Mann-Whitney U test; BDNF: Brain-derived neurotrophic factor; HbA1c: Hemoglobin A1c

Characteristics	Case (With Diabetic Retinopathy) (n=44)	Control (Without Diabetic Retinopathy) (n=44)	P value*
Age (years)	51.8 ± 9.446	53.36 ± 10.49	0.4633
Male/female**	28/16	17/27	<0.05
Duration of diabetes (months)	123.7 ± 77.19	52.53 ± 53.49	<0.0001
Systolic blood pressure (mmHg)	136.3 ±13.27	135.2 ±13.33	0.7133
Diastolic blood pressure (mmHg)	82.95 ± 9.981	83.7 ± 9.065	0.66
Serum HbA1c (%)	8.502 ± 2.105	8.075 ± 2.092	0.3422
Fasting blood glucose (mg/dl)	135.9 ± 52.81	128.6 ± 48.25	0.497
Post-prandial blood sugar (mg/dl)	221.6 ± 86.3	188.2 ± 57.36	0.03
Blood urea nitrogen (mg/dl)	27.58 ± 13.81	22.46 ± 9.27	0.04
Serum creatinine (mg/dl)	1.09 ± 0.43	0.87 ± 0.15	0.002
Serum BDNF (pg/ml)***	1598 ± 483	2753 ± 465	<0.0001

**Figure 1:**
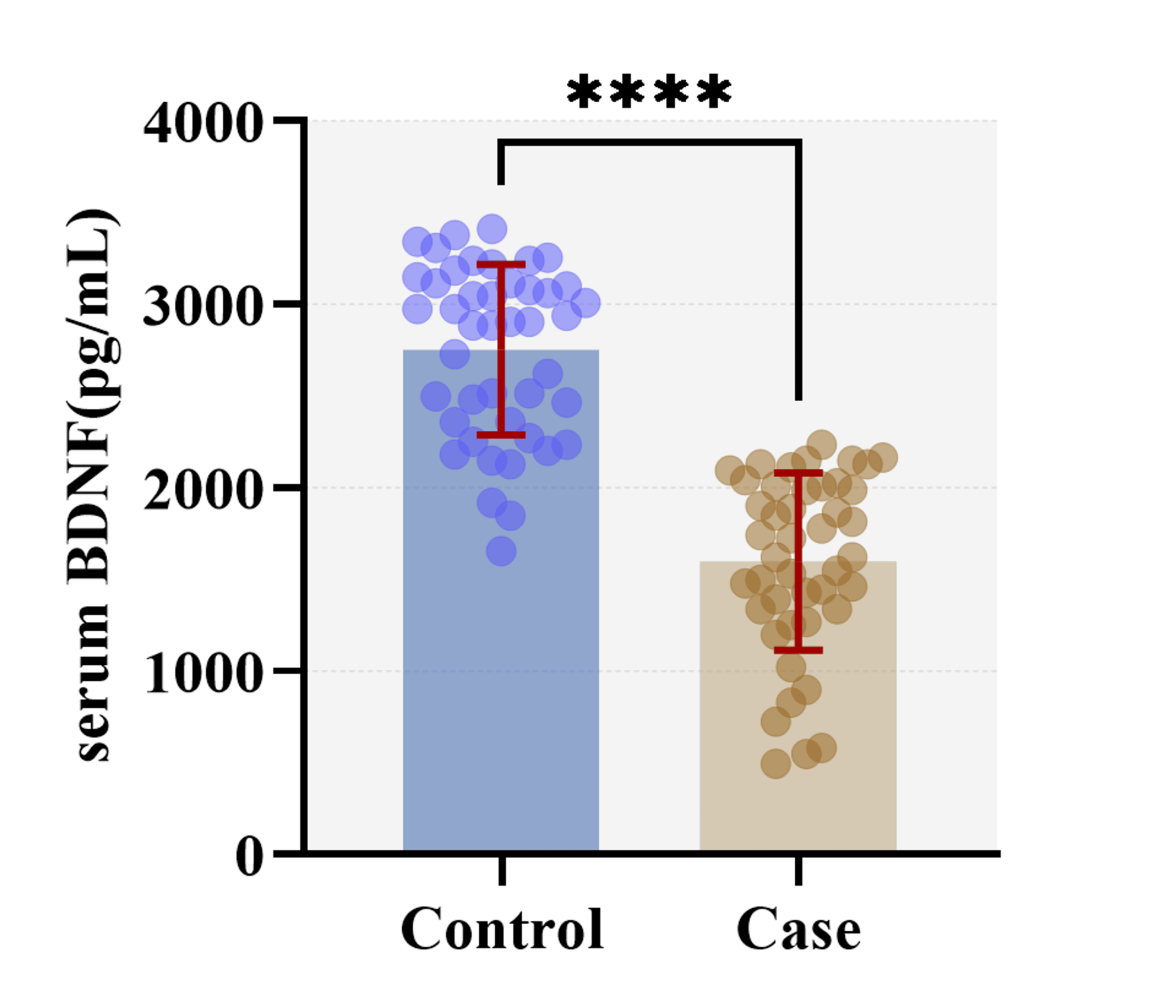
Comparison of serum BDNF levels between the groups (control and case) using Mann-Whitney U test (U=64, p<0.0001). BDNF: Brain-derived neurotrophic factor

The mean serum BDNF in the control and case groups was 2753 ± 465 pg/ml and 1598 ± 483 pg/ml, respectively (Figure 1). The difference between the two groups was found statistically significant by the Mann-Whitney U test (U= 64, p<0.0001). The minimum serum BDNF concentration was 495 pg/ml and 1655 pg/ml in the case and control groups, respectively. The maximum BDNF concentration was 2235 pg/ml and 3413 pg/ml in the case and control groups, respectively. Mean concentrations of serum BDNF were compared in various grades of DR. The mean BDNF was found to be minimum in the moderate non-proliferative diabetic retinopathy (NPDR) group (1517.02 ± 513.74), whereas it was found to be maximum in the severe NPDR group (1719.76 ± 308.50). The differences in the mean between these groups were compared using the Kruskal-Wallis test, which was found to be statistically insignificant (H=0.5564, p=0.906). The mean BDNF levels in male and female groups were 2042.80 ± 736.1 pg/ml and 2320.78 ± 743.2 pg/ml, respectively. However, comparing the groups with the Mann-Whitney U test, there was no statistically significant difference between the two groups (U=751.5, p=0.0735).

**Figure 2:**
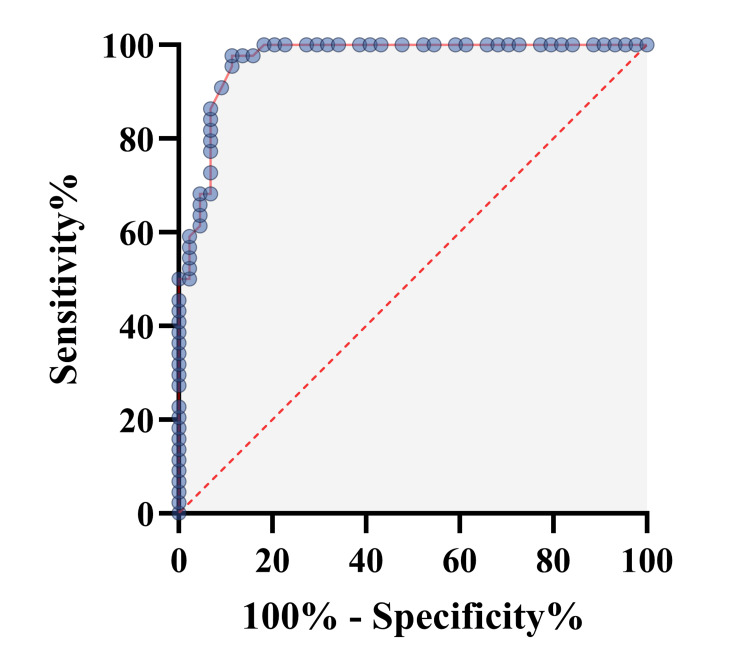
ROC curve for serum BDNF showing that BDNF is a strong biomarker that differentiates the case and control groups with AUC of 0.967 (95% CI 0.933-1, p<0.0001). The cut-off value is 2139 pg/ml with sensitivity and specificity of 90.91%. ROC: Receiver operating characteristic; AUC: Area under curve; BDNF: Brain-derived neurotrophic factor

Based on the receiver operating characteristic (ROC) curve (Figure 2), the optimal cut-off value of serum BDNF level as an indicator for diagnosis of DR was projected to be 2139 pg/ml, which yielded a sensitivity of 90.91% and a specificity of 90.91%, with the area under the curve as 0.967 (95% CI 0.933-1, p<0.0001).

Univariate and multivariate logistic regression analysis was performed to study the effect of various predictors with regard to DR. The above table shows that BDNF is a good predictor for DR after multivariate regression. For each 1 pg/ml increase in plasma level of BDNF, the unadjusted and adjusted risk of DR would be decreased by 0.7% (with odds ratio (OR) of 0.993, CI=0.988-0.996, p=0.002) and 0.8% (with OR of 0.992, CI=0.987-0.997, p=0.0015), respectively.

## Discussion

Diabetes is a long-term metabolic disorder marked by high blood sugar levels, which over time can cause significant harm to the heart, blood vessels, eyes, kidneys, and nerves. According to the most recent data from the International Diabetes Federation, 537 million adults were living with diabetes in 2021, and this number is projected to increase to 643 million by 2030 and 783 million by 2045. Three-fourths of the people with diabetes live in low and middle-income countries. Over half of the patients with diabetes remain undiagnosed. Diabetes caused 6.7 million deaths in 2021 [1]. Taking into consideration the large prevalence of DM and its associated complications, it has been one of the most studied areas in medical research. So we took up this study to determine the association of serum levels of BDNF with DR.

The association of serum BDNF with DR is the least studied topic; the few available literature give contradictory findings that direct the need for further research. Our study was a hospital-based case-control study. The study included 88 subjects divided into two groups - diabetic patients with retinopathy (case) and diabetic patients without retinopathy (control). Estimation of serum BDNF levels was done by ELISA. The data were entered and analyzed using GraphPad Prism software. Demographic details like age and gender distribution were studied. Blood sugar levels - fasting and postprandial - and HbA1c levels were studied. Duration of diabetes and history of hypertension in study subjects were also analyzed. Other biochemical parameters that were taken up for study included serum urea, creatinine, total cholesterol, low-density lipoprotein (LDL), and triglycerides. We investigated serum BDNF levels and their correlation with the above-mentioned clinical and biochemical parameters.

As per our study, the mean serum BDNF in the control and case groups was 2753 ± 465 pg/ml and 1598 ± 483 pg/ml, respectively. Serum BDNF levels were significantly higher in the control group. The ROC curve analysis for serum BDNF identified an optimal cut-off level of 2139 pg/ml for diagnosing DR. At this level, both sensitivity and specificity were 90.91% with an area under the curve of 0.967 (95% CI 0.933-1, p<0.0001). However, in a study by Liu et al., the ROC curve yielded a cut-off value of 13.6 ng/ml for the diagnosis of DR, with a specificity of 70.2% and a sensitivity of 80.5% [9]. Boyuk et al. carried out a study in Turkey comparing serum BDNF levels between type 2 DM patients and healthy controls. The ROC analysis identified a cut-off of >137 pg/ml as indicative of type 2 DM, with a sensitivity of 71.79% and a specificity of 68% [10]. The difference in the ROC curves obtained in these studies can be attributed to several factors including variations in study populations and methodologies, particularly differences in the ELISA kits used.

The average serum BDNF levels across groups categorized by DR grading were compared using the Kruskal-Wallis test, revealing no statistically significant difference between the groups (H=0.5564, p=0.906). This lack of significance might be attributed to the unequal distribution of study participants across the groups. A study by Rashid et al. in Lahore, Pakistan, showed a statistically relevant difference in serum levels of BDNF among study groups (p<0.001) [11]. Ola et al. did a similar study in Saudi Arabia. The mean serum levels of BDNF in the control group, diabetic patients without retinopathy, and proliferative diabetic retinopathy (PDR) patients were 25.5 ± 8.5, 21.8 ± 4.9, and 10.01 ± 8.1 ng/ml, respectively. They found a statistically relevant difference between those without retinopathy and the PDR group [12].

The mean BDNF levels in male and female groups were 2042.80 ± 736.1 pg/ml and 2320.78 ± 743.2 pg/ml, respectively. Though statistically insignificant, the higher mean value in females may account for the uneven gender ratio between the study groups. More females were included in the control group, whereas males occupied a larger portion of the case group. However, in a study by Liu et al., the plasma BDNF levels were relatively higher in women patients as compared to men (p<0.001) [9].

Logistic regression analysis in our study inferred for each 1 pg/ml increase in plasma level of BDNF, the unadjusted and adjusted risk of DR would decrease by 0.7% (with OR of 0.993, CI=0.988-0.996, p=0.002) and 0.8% (with OR of 0.992, CI=0.987-0.997, p=0.0015), respectively. Consistent with our findings, a negative association between BDNF and DR was obtained in studies by Taşlipinar Uzel et al. (2020), Liu et al. (2016), Kaviarasan et al. (2015), and Jun et al. (2021) [5,9,13,14]. Contradictory findings of a positive association of serum BDNF in diabetes were obtained by Boyuk et al. (2014) and Suwa et al. (2006) [10,15].

A wide disparity in serum BDNF values was noticed in different studies. Davarpanah et al. conducted a systematic review and meta-analysis on the association between BDNF and type 2 diabetes, which revealed the values ranging from 0.37 to 31 ng/ml in type 2 DM patients and 0.13 to 39 ng/mL in healthy controls across different studies [16]. The inconsistencies among studies may be attributed to differences in assay techniques, sample testing methods, subject populations, and analytic platforms. Additionally, variability in serum BDNF measurements could result from factors such as the time of day blood samples that were collected, as well as differences in processing and storage methods.

Lastly, we would like to point out certain strengths and limitations of our study. It is the first time a study has been done on the association of serum BDNF with DR in this part of the country. Also, the available literature tells of the significant role of BDNF in diabetes and other diseases. So it is an appropriate time to do extensive research to explore the role of BDNF, be it diagnostic or therapeutic. Coming to the limitations, our study was done on 88 subjects, a relatively small sample size for generalizing the findings to a broader population. Also, most of the patients we had were from rural backgrounds, and it was difficult to get them convinced to be a part of our research. As a hospital-based case-control study, it inherently has limitations in establishing causality. To determine a causal relationship, longitudinal studies need to be conducted. Longitudinal data would be more informative in understanding the dynamics of BDNF levels with DR. Also, our study exclusively focussed on BDNF. DR is a condition influenced by multiple biomarkers not examined in our study. To cover up these lacunae, we hope to do further research on BDNF in the coming years.

## Conclusions

In a nutshell, our study concludes that serum BDNF has a statistically significant difference between the study groups and could be used as a predictive marker for DR. We noticed some discrepancies between our study and previously conducted research, highlighting the need for further research on this topic. As far as we are concerned, research on BDNF in diabetes has hardly been explored in an Indian scenario, which adds a unique aspect to this research.
